# Tumor-Suppressive Function of miR-139-5p in Esophageal Squamous Cell Carcinoma

**DOI:** 10.1371/journal.pone.0077068

**Published:** 2013-10-18

**Authors:** Ran Liu, Miao Yang, Yanli Meng, Juan Liao, Jingyi Sheng, Yuepu Pu, Lihong Yin, Sun Jung Kim

**Affiliations:** 1 Key Laboratory of Environmental Medicine Engineering, Ministry of Education, School of Public Health, Southeast University, Nanjing, China; 2 North China Petroleum Bureau General Hospital, Renqiu, China; 3 Department of Life Science, Dongguk University, Seoul, Korea; Sanjay Gandhi Medical Institute, India

## Abstract

Recent studies have demonstrated the possible function of miR-139-5p in tumorigenesis. However, the exact mechanism of miR-139-5p in cancer remains unclear. In this study, the association of miR-139-5p expression with esophageal squamous cell carcinoma (ESCC) was evaluated in 106 pairs of esophageal cancer and adjacent non-cancerous tissue from ESCC patients. The tumor suppressive features of miR-139-5p were measured by evaluating cell proliferation and cell cycle state, migratory activity and invasion capability, as well as apoptosis. Luciferase reporter assay and Western blot analysis were performed to determine the target gene regulated by miR-139-5p. The mRNA level of NR5A2, the target gene of miR-139-5p, was determined in ESCC patients. Results showed that reduced miR-139-5p level was associated with lymph node metastases of ESCC. MiR-139-5p was investigated to induce cell cycle arrest in the G0/G1 phase and to suppress the invasive capability of esophageal carcinoma cells by targeting the 3′UTR of oncogenic NR5A2. Cyclin E1 and MMP9 were confirmed to participate in cell cycle arrest and invasive suppression induced by NR5A2, respectively. Pearson correlation analysis further confirmed the significantly negative correlation between miR-139-5p and NR5A2 expression. The results suggest that miR-139-5p exerts a growth- and invasiveness-suppressing function in human ESCCs, which demonstrates that miR-139-5p is a potential biomarker for early diagnosis and prognosis and is a therapeutic target for ESCC.

## Introduction

MicroRNAs (miRNAs), which are 18 nt to 24 nt non-coding RNAs, are suggested to serve important roles in cell proliferation, differentiation, apoptosis, and development [1], [2], [3]. Over 2042 miRNAs have been identified in humans and are known to regulate the activity of more than a third of human genes either by complete or partial complementary binding to specific sequences of target mRNAs, which subsequently induce mRNA degradation or translational inhibition [4], [5], [6]. Many studies have demonstrated that disruption of microRNA function may be crucial in the disease process, particularly in cancer oncogenesis [7], [8], [9], [10].

Esophageal cancer ranks eighth in order of occurrence and sixth as the leading cause of cancer mortality worldwide [11]. China has a high risk and mortality rate from esophageal squamous cell carcinoma (ESCC). The overall five-year survival rate is poor, given that most cases of ESCC could not be diagnosed until the disease is at the advanced stages [12], [13]. Therefore, identifying tissue-specific biomarkers is an important strategy for the early diagnosis of ESCC. A differential expression profile of miRNAs was identified using miRNA chip technology in esophageal cancer and adjacent non-cancerous tissue of three newly diagnosed ESCC patients from Huaian in our laboratory, one of the areas with the highest ESCC morbidity in China. Consistent with the results from previous studies, the expression of miR-139-5p was found to be significantly reduced in esophageal cancer tissues, thereby implicating its tumorigenic relevance in multiple cancer types [14], [15], [16].

In this study, the relationship between miR-139-5p expression and the development of esophageal cancer was further validated in 106 pairs of esophageal cancer tissues and adjacent normal tissues. To investigate the potential functional roles of miR-139-5p in esophageal carcinogenesis, miR-139-5p expression was analyzed in five ESCC cell lines, and a miR-139-5p mimic treatment of an ESCC cell line was established for genome-wide microarray analysis. The tumor-suppressive features of miR-139-5p in vitro, including cell proliferation and cell cycle involvement, migratory activity and invasion, as well as apoptosis, were then explored. Moreover, the potential mRNA target of miR-139-5p, nuclear receptor subfamily 5, group A, member 2 (NR5A2), which induces cell proliferation through the concomitant induction of cyclin D1 and E1 and promotes cancer invasion through the remodeling of the actin cytoskeleton and E-cadherin cleavage in cancer development and metastasis [17], [18], [19], [20], [21], was identified using 3′UTR luciferase reporter assay and correlation analysis with miR-139-5p expression in a population study, which provides insight into the mechanisms underlying miRNA deregulation in esophageal cancer.

## Results

### Demographic Characteristics

The average age of 106 patients with newly diagnosed, untreated ESCC was 61.68±7.84 years. The male-to-female ratio was 2.21. Seventy-eight of 106 patients (73.6%) were diagnosed as well differentiated (I+II), whereas 28 patients (26.4%) were diagnosed as poorly differentiated. Thirty-four of 106 patients (32.1%) were diagnosed to have lymph node metastases.

### Differential Expression of miR-139-5p in ESCC Tissues and Adjacent Normal Tissues

Table 1 presents the relative expression levels of miR-139-after normalization with U6. Significant differences in miR-139-5p expression between cancer tissues and adjacent normal tissues were observed, and the average fold change decrease of miR-139-5p in cancer tissues was 14.065 compared with adjacent normal tissues. Conditional logistic regression analysis revealed that reduced miR-139-5p expression was highly associated with increased risk for esophageal cancer (OR = 2.024). Further analysis of miR-139-5p association with clinicopathological features showed that miR-139-5p expression levels were significantly reduced in patients with lymph node metastasis compared with those without lymph node metastasis (P = 0.040, Table 2).

**Table 1:** Downregulation of miR-139-5p expression associated with a high risk for ESCC.

Group	Size	Relative expression level(Mean, 95%CL)	Differential expressionlevel (Mean, 95%CL)	P value (t test)	OR (95%CL)
Tumor tissues	106	−9.027 (−15.651, −2.261)	−3.814 (−11.591, 3.963)	<0.0001	0.494 (0.382, 0.639)
Adjacent normal tissues	106	−5.212 (−11.339, 1.040)			

**Table 2:** Associations of miR-139-5p expression with demographic and clinical characteristics.

Group	Size	Differential expression level (Mean, 95%CL)	P value
**Age**			
<60	44	−3.842 (−4.998, −2.686)	0.952
≥60	62	−3.795 (−4.839, −2.751)	
**Gender**			
male	73	−4.065 (−5.010, −3.121)	0.336
female	33	−3.260 (−4.602, −1.918)	
**Family history of cancer**			
No	90	−3.652 (−4.442, −2.863)	0.320
Yes	16	−4.728 (−7.384, −2.073)	
**Tumor location**			
Upper part of esophagus	6	−4.835 (−7.075, −2.594)	0.434
Middle part of esophagus	89	−3.596 (−11.822, 4.630)	
Lower part of esophagus	11	−5.024 (−10.202, 0.154)	
**Differentiation**			
I and II	78	−3.805 (−4.671, −2.938)	0.966
III	28	−3.842 (−5.536, −2.148)	
**Lymph node metastasis**			
No	72	−3.138 (−3.810, −2.466)	0.040
Yes	34	−5.246 (−7.138, −3.355)	
**Smoking index**			
<400	45	−3.617 (−4.639, −2.595)	0.662
≥400	61	−3.960 (−5.077, −2.844)	
**Alcohol use**			
None or occasional	55	−4.527 (−5.726, −3.328)	0.054
Often	51	−3.046 (−3.964, −2.128)	

### Genome-wide Transcription Network Analysis Affected by Overexpressed miR-139-5p

The relative expression of miR-139-5p in miR-139-5p mimic-treated EC109 cells using quantitative reverse transcription PCR (qRT-PCR) analysis normalized by U6 expression showed a significant increase of 627-fold compared with negative control (NC) oligo-treated cells. To identify the function of miR-139-5p in esophageal cancer oncogenesis, the above two total RNA samples were used for the genome-wide expression microarray assay. Clustering analysis showed good reproducibility between the parallel samples and could successfully distinguish between the treatment group from the NC group. Eighty-nine upregulated (ratio>2) and 265 downregulated mRNAs (ratio<0.5) were identified from miR-139-5p mimic-treated cells relative to NC cells (Table S1). All microarray data were uploaded to the Gene Expression Omnibus database (accession # GSE42486).

**Figure 1:**
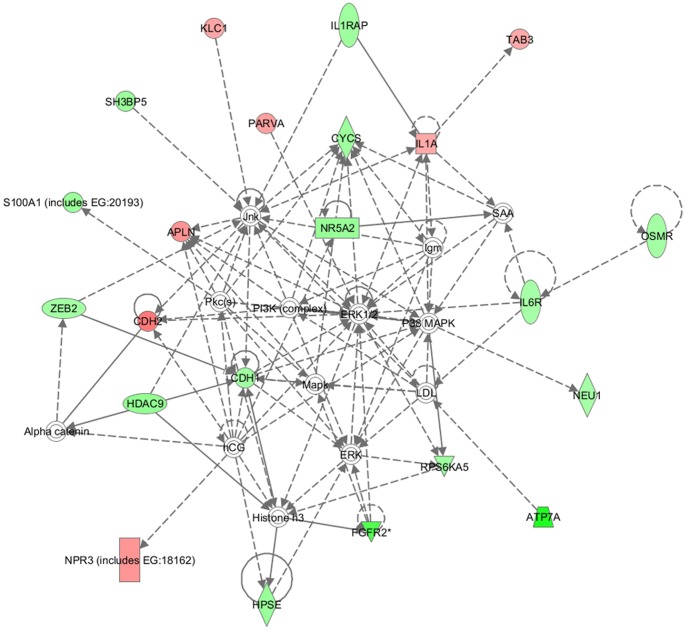
The top interactional network of altered genes following the miR-139-5p mimic transfection. The top interactional network was identified by the Ingenuity Pathway Analysis software as significant function association of differentially expressed genes following introduction of miR-139-5p mimic. The set of input genes matched with Ingenuity Pathways Knowledge Base and their fold change value were used to identify hypothetical interaction models of genes. According to the manually created Ingenuity Pathway Analysis database, the network had relevance for “Cellular Movement, Lipid Metabolism, and Small Molecule Biochemistry”.

Ingenuity pathway analysis (IPA) was performed to determine the biological functions that could be influenced by the overexpressed miR-139-5p in the EC109 cell line with the identified transcripts. A list of the top 10 networks significantly associated with the input genes is available in Table S2. The most significant functions associated with the altered genes include cellular movement, cellular growth and proliferation, cell death, and cancer. The top interactional network identified was designated by IPA as having relevance for “Cellular Movement, Lipid Metabolism, and Small Molecule Biochemistry” (Score = 33, Figure 1). Investigating the transcripts for disease-relevance yielded a high number of interactions that were pertinent to cancer and cancer-related processes involving 73 differential molecules (P = 1.2E-04). According to molecular and cellular functions, 24 of molecules involved in cellular growth and proliferation process were directly or indirectly regulated by miR-139-5p (P = 3.73E-03), whereas 13 and 12 of the molecules were respectively associated with cell death and cell cycle progression (P = 2.77E-03 and 3.66E-03).

### miR-139-5p Suppresses Cell Proliferation Ability in vitro

**Figure 2:**
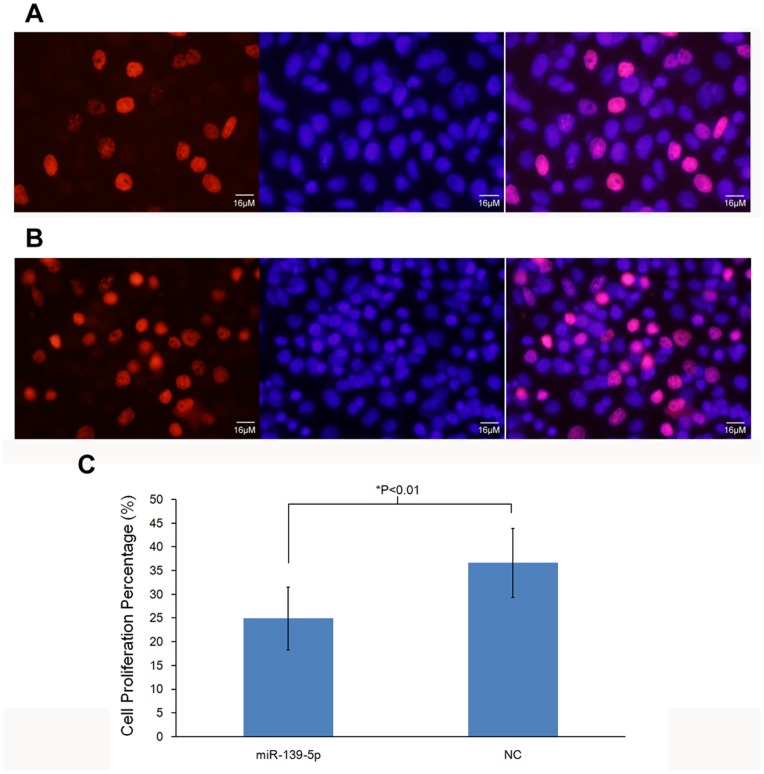
EdU DNA cell proliferation assay of EC109 cells induced by miR-139-5p mimic. EC109 cells were treated with miR-139-5p mimic (A) or negative control (B). The left image shows proliferating cells labeled with EdU. The middle one shows a total population of cells, in which nucleic acid were stained with Hoechst 33342. The right one represents the incorporated image in which the proliferating cells present light points. C represents the cell proliferation percentage of proliferating cells out of total population. The figures show significantly reduction (P<0.01) in the proliferation rate in EC109 cells treated with miR-139-5p mimic.

EdU DNA Cell Proliferation assay was used to determine the impact of miR-139-5p on the cell proliferation of EC109 cells. A 2 h pulse of EdU was found to label approximately one-third of the control cells, as expected for a rapidly dividing population (Figures 2A and 2C), but it only labeled approximately one-fourth of cells that were transfected with miR-139-5p mimic (Figures 2B and 2C). The results revealed that the overexpression of miR-139-5p significantly suppressed the cell proliferation rate of EC109 cells (P<0.01).

### Upregulated miR-139-5p Expression Arrests Cells in G0/G1 Phase

**Figure 3:**
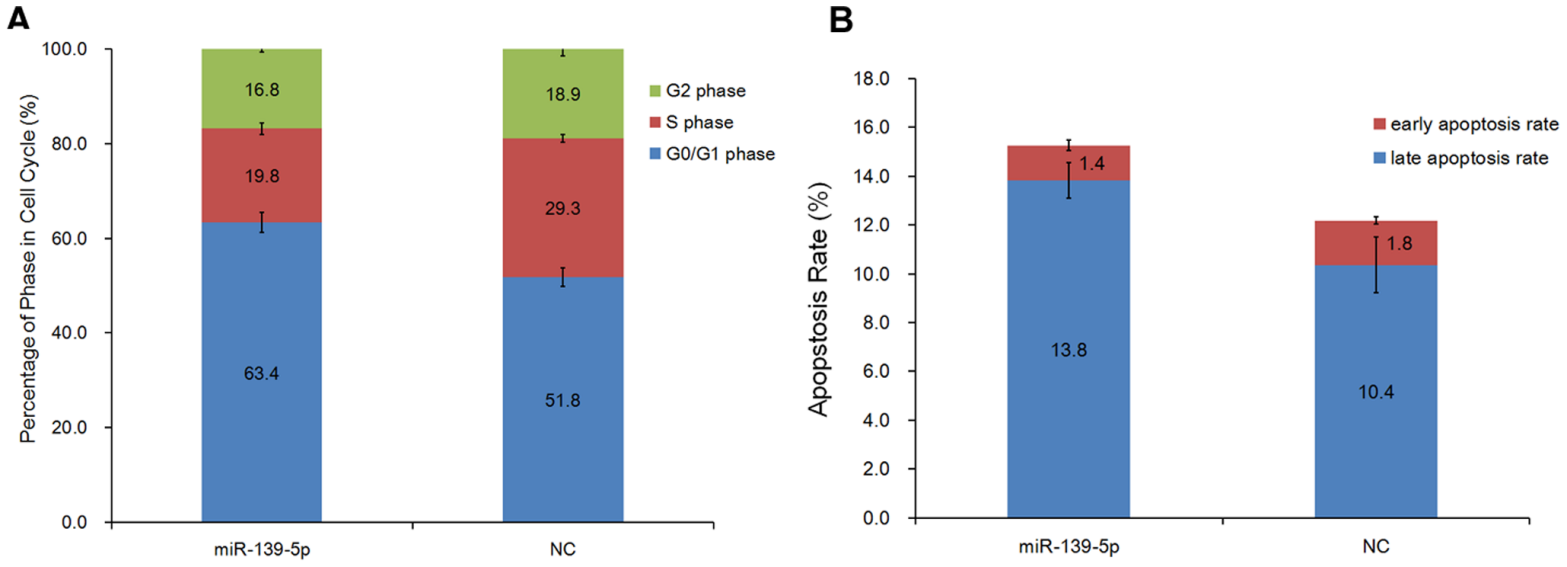
Cell cycle and apoptosis assay of miR-139-5p overexpression in EC109 cells. (A) FACS analysis was performed to determine the constitution of cell cycle in miR-139-5p transfected cells and negative control cells. The cell percentage at different phases showed a cell cycle arrest in G0/G1 phase when treated with miR-139-5p mimic (P<0.05). (B) The apoptosis rate of EC109 cells transfected with miR-139-5p or negative control was determined using Annexin V-FITC and PI double staining. The late apoptosis rate in cells transfected with miR-139-5p mimic was significantly increased compared with negative control cells (P<0.05).

Flow cytometry was used to determine the effect of miR-139-5p on the cell cycle phase of EC109. As shown in Figure 3A, for miR-139-5p transfected cells, the constituent ratio of cell cycle phases significantly differed from that in NC cells (x^2^ = 45.70, P<0.0001). The G0/G1 phase cell population in miR-139-5p transfected cells increased compared with NC cells, accompanied by a decrease of cells in the S phase. The results showed a cell cycle arrest in the G0/G1 phase upon treatment with miR-139-5p mimic, suggesting that miR-139-5p participates in the regulation of the G1/S phase transition during cell cycle progression.

### miR-139-5p Induces Late Apoptosis of ESCC Cells in vitro

The apoptosis rate of EC109 cells transfected with miR-139-5p or NC using Annexin V-FITC and PI double staining was simultaneously determined. As shown in Figure 3B, the early apoptosis rate was similar between miR-139-5p treatment and NC group, whereas the late apoptosis rate in cells transfected with miR-139-5p mimic was significantly increased compared with that in NC cells (P<0.05). The results reveal that miR-139-5p overexpression induced the late apoptosis of EC109 cells.

### Overexpression of miR-139-5p Suppresses Cell Migratory Activity and Cell Invasiveness in vitro

**Figure 4:**
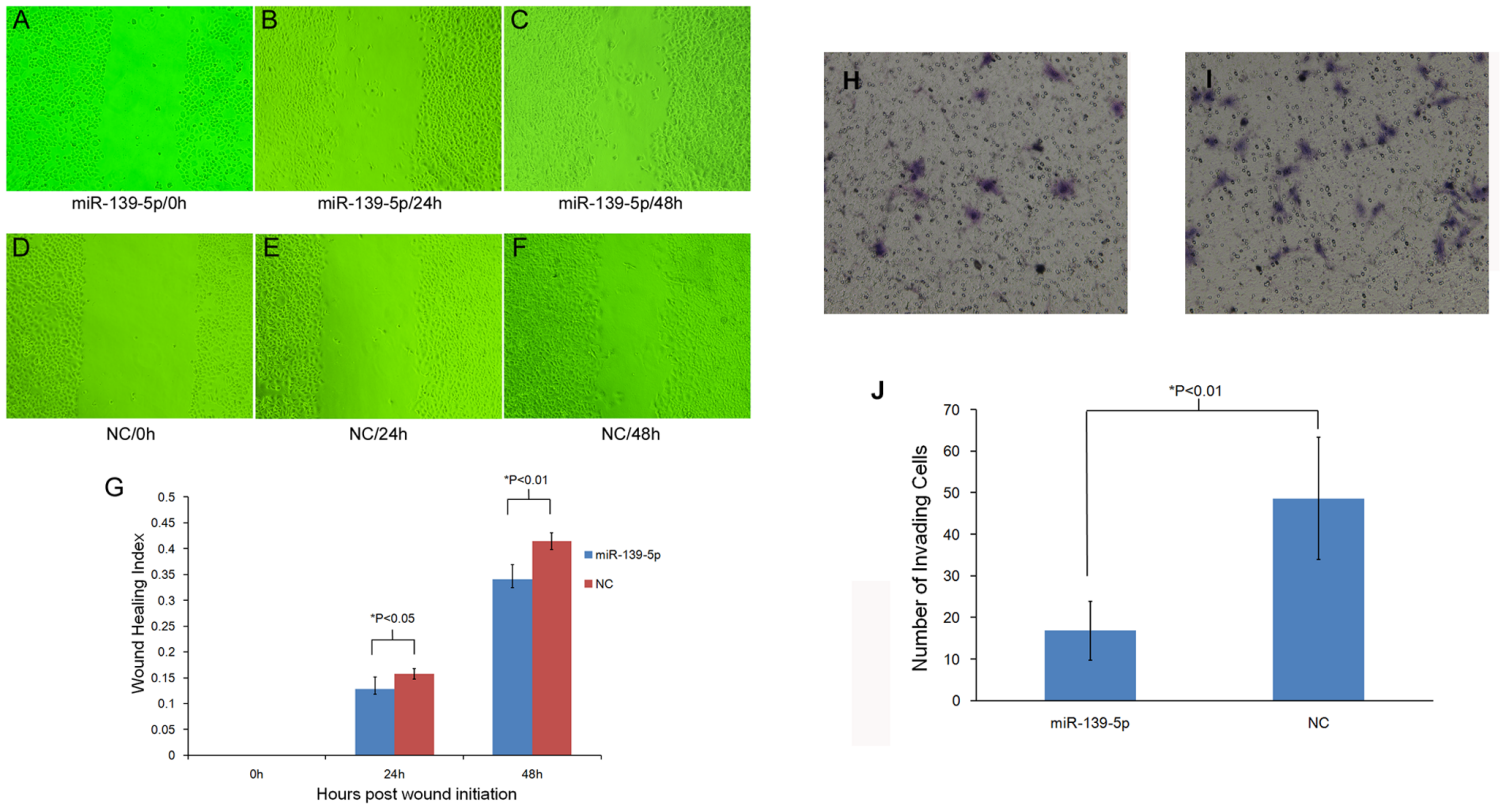
Cell migratory activity and invasive ability assay of EC109 cells induced by miR-139-5p. A to F shows the effect of miR-139-5p transfection on cell migratory activity using cell scratch experiment. Wound healing was measured at 0, 24, and 48 h after wounding in miR-139-5p treated cells (A, B, and C, respectively) and negative control (D, E, and F, respectively). G represents quantitation of migration following miR-139-5p transfection versus controls. The wound healing index was defined as the ratio of (initial wound width-wound width after healing at specific time) divided by initial wound width. The result showed that miR-139-5p over-expression significantly suppressed cell migration both at 24 h and 48 h after wounding. Cell invasive ability of EC109 cells treated with miR-139-5p mimic (H) or negative control (I) was determined using a 24-well transwell plate. The invading cells transferred to the underside of the transwell membrane were stained with hematoxylin-eosin for counting. J represents the number of invading cells following miR-139-5p transfection versus controls. The results showed that the number of invading cells when treated with miR-139-5p mimic is significantly reduced compared with negative control (P<0.01).

To assess the effect of miR-139-5p on cell migratory and invasive capability, cell scratch experiment and transwell invasive experiment were respectively conducted. The cell scratch experiment showed a significant difference between miR-139-5p treatment and NC at either the 24 h or 48 h time points after wounding (Figures 4A to 4G). To exclude the effect of cell proliferation on the evaluation of cell migratory activity, a transwell invasive experiment was performed, and the results showed that the invading cells on the underside of the membrane were significantly reduced when transfected with miR-139-5p mimic compared with those from NC group (Figures 4H to 4J, P<0.01). The results imply that miR-139-5p not only affects cell migratory activity, but also suppresses the invasive capability of EC109 cells.

### miR-139-5p Regulates NR5A2 Protein and mRNA Level

**Figure 5:**
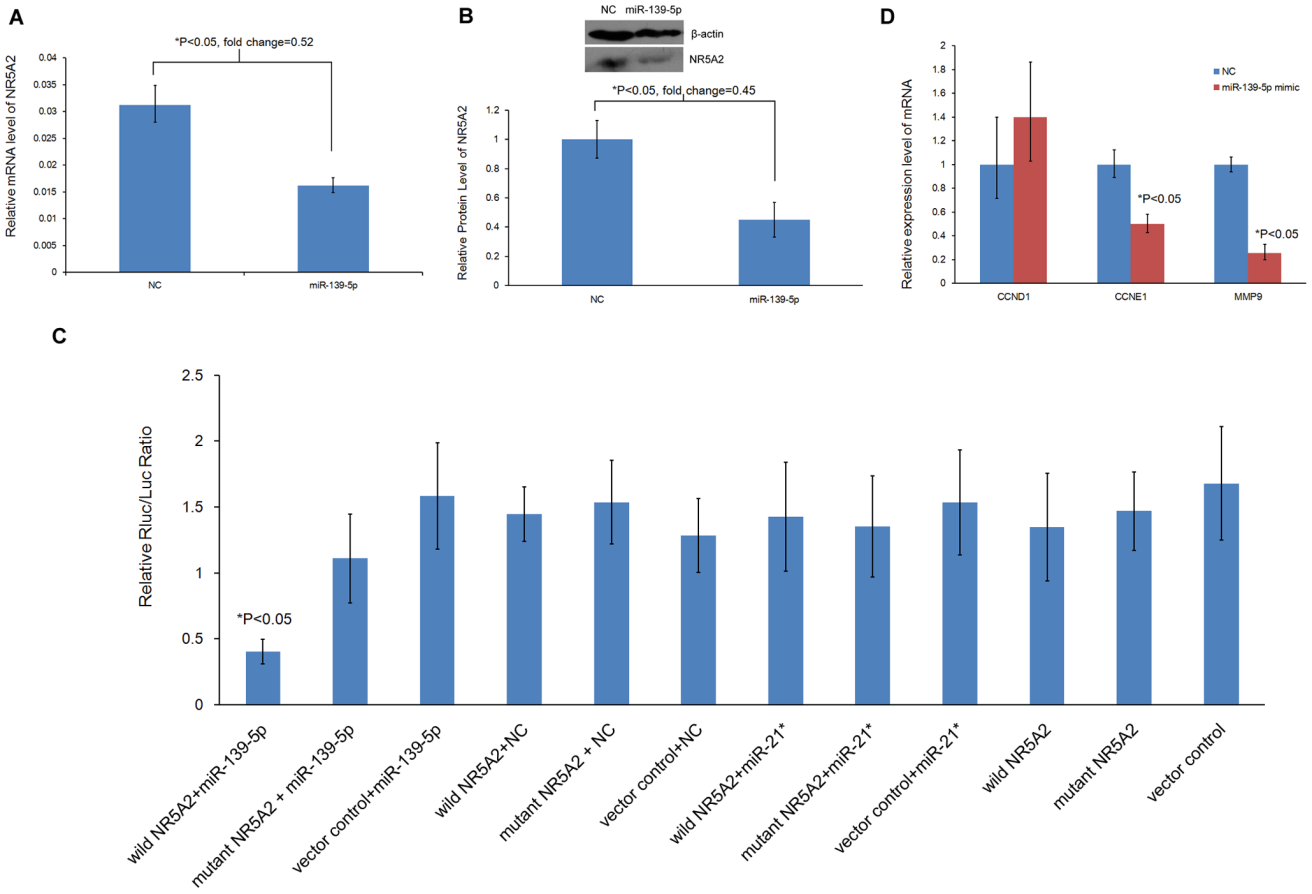
Identification of NR5A2 regulated by miR-139-5p and downstream genes. A shows relative expression of NR5A2 in EC109 cells treated with miR-139-5p mimic or negative control normalized against β-actin using quantitative real time RT-PCR analysis. B represents the protein level of NR5A2 in EC109 cells treated with miR-139-5p mimic or negative control normalized against β-actin using Western blot assay. C shows luciferase reporter assay for 3′UTR of NR5A2 regulated by miR-139-5p. Bar represents the luciferase signal ratio for Renilla over Firefly of miR-139-5p mimic or controls co-transfection with 3′UTR constructs of NR5A2 or control. Negative control (NC) and miR-21* represent the negative and non-specific control of miR-139-5p, respectively. Vector control and mutant NR5A2 represent the negative and non-target binding site control of 3′UTR construct of NR5A2, respectively. An asterisk (*) indicates a statistically significant difference. The results show that the mRNA and protein levels of NR5A2 were significantly reduced in EC109 cells when transfected with the miR-139-5p mimic. Luciferase reporter assay indicates that miR-139-5p could suppress NR5A2 expression by binding to specific 3′UTR sequences. D represents the relative expression levels of cyclin D1, cyclin E1, and MMP9 mRNA associated with cell cycle arrest and cell invasiveness in miR-139-5p mimic-transfected EC109 cells (red bar) compared with negative control cells (blue bar) using quantitative reverse transcription PCR. After normalization against β-actin, the mRNA levels of cyclin E1 and MMP9 were significantly reduced in EC109 cells transfected with the miR-139-5p mimic (P<0.05).

Nuclear receptor subfamily 5, group A, member 2 (NR5A2), a gene associated with cell cycle and cancer invasion, was predicted to harbor an 8-mer site that matches the complementary sequence of the miR-139-5p seed region based on a miRNA target prediction tool (TargetScan Release 6.0). To investigate whether miR-139-5p affects NR5A2 expression, the protein and mRNA levels of NR5A2 in EC109 cells were measured after transfection with miR-139-5p mimic using Western blot analysis and qRT-PCR. After normalization by β-actin, the mRNA and protein levels of NR5A2 were significantly reduced in EC109 cells transfected with miR-139-5p mimic (P<0.05), as shown in Figures 5A and 5B. The results indicate that miR-139-5p suppressed NR5A2 expression, acting as a post-transcriptional repressor.

### miR-139-5p Suppresses NR5A2 Expression by Binding to 3′UTR Sequence

To validate the mechanism of NR5A2 regulation by miR-139-5p, luciferase reporter assay was performed by transfecting the 3′UTR of NR5A2 constructs. Figure 5C shows the luciferase signals from co-transfection of miR-139-5p mimics or NC with wild-type or mutant of 3′UTR constructs of NR5A2. NC and miR-21* are the negative and non-specific controls of miR-139-5p, respectively. Vector control and mutant NR5A2 are the negative and non-target binding site controls of 3′UTR construct of NR5A2, respectively. ANOVA analysis indicated that the miR-139-5p mimic significantly inhibited the expression of wild 3′UTR constructs of NR5A2 (P<0.05) compared with other treatments. Moreover, co-transfection of miR-139-5p mimics with wild-type 3′UTR constructs of NR5A2 showed a 2.7-fold decrease in NR5A2 expression compared with the mutant type of 3′UTR constructs of NR5A2. The results suggest that miR-139-5p could suppress NR5A2 expression by binding to specific 3′UTR sequence.

### miR-139-5p Affects Cell Cycle and Invasiveness through Cyclin E1 and MMP9 Regulated by NR5A2

To investigate whether miR-139-5p affects cell cycle and invasiveness through downstream genes regulated by NR5A2 in esophageal cancer, the mRNA levels of cyclin D1 and cyclin E1 were determined during cell cycle arrest, and the mRNA of MMP9 was detected during cell invasiveness in EC109 cells after transfection with miR-139-5p mimic using qRT-PCR. As shown in Figure 5D, after normalization by β-actin, the mRNA levels of cyclin E1 and MMP9 were significantly changed in EC109 cells after transfection with the miR-139-5p mimic (P<0.05). The results indicate that as the downstream genes regulated by NR5A2, cyclin E1 and MMP9 respectively participated in the cell cycle arrest and invasive suppression induced by miR-139-5p.

### Association of NR5A2 mRNA Level with miR-139-5p Expression in ESCC Cell Lines and Esophageal Tissues

**Figure 6:**
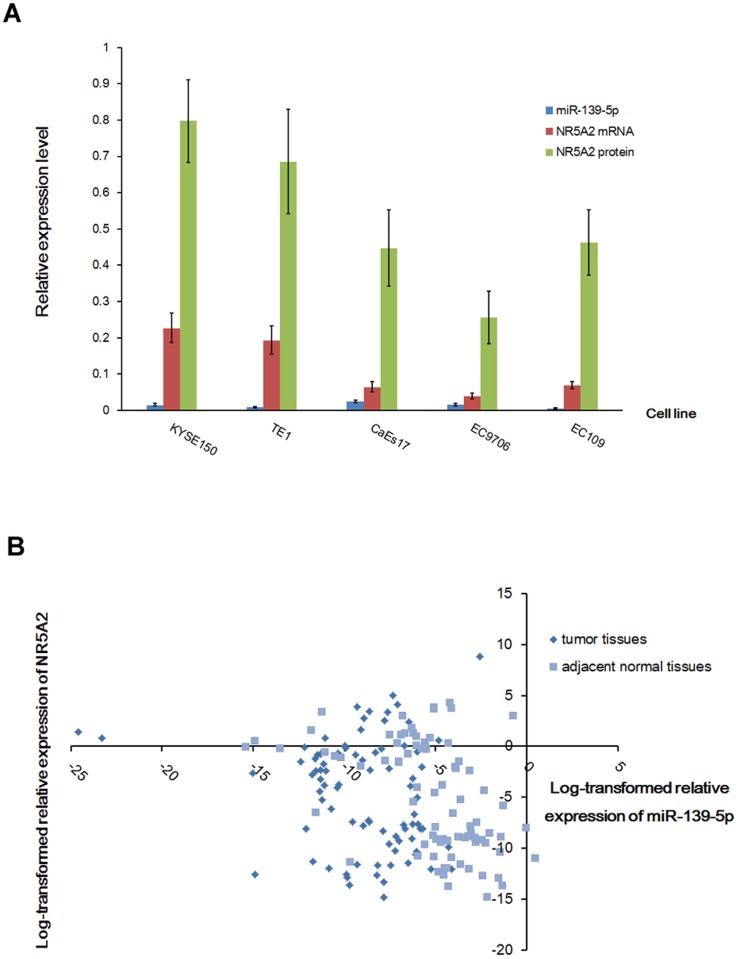
Association of miR-139-5p and target gene NR5A2 in ESCC cell lines and ESCC patients. A shows the relative expression of miR-139-5p and NR5A2 in five ESCC cell lines. Blue bar represents the relative expression of miR-139-5p normalized against U6 in five ESCC cell lines. Red and green bar represents the relative mRNA and protein level of NR5A2 normalized against β-actin in five ESCC cell lines, respectively. Correlation analysis of the miR-139-5p expression and NR5A2 showed moderate correlation in four ESCC cell lines except for KYSE-150 (R = -0.398 for NR5A2 mRNA, and R = −0.370 for NR5A2 protein, respectively). B shows the scatter plot of miR-139-5p and NR5A2 expression in ESCC patients. Relative expression of miR-139-5p and NR5A2 that were normalized against U6 and β-actin, respectively, were evaluated using Pearson correlation analysis. The mixed sample set, including tumor tissues (diamond) and adjacent normal tissues (square) from ESCC patients, showed a significantly negative correlation between miR-139-5p and NR5A2 expression (R = −0.284, P = 0.0002).

To explore the actual association between NR5A2 and miR-139-5p, the relative expression of NR5A2 mRNA and miR-139-5p was determined in five ESCC cell lines. As shown in Figure 6A, EC109 showed the lowest expression of miR-139-5p, and CaEs-17 had the highest expression, whereas NR5A2 showed the highest expression in KYSE-150 but the lowest expression level in EC9706. Considering that KYSE-150 is an anti-radiation cell line established from a poorly differentiated esophageal squamous cell carcinoma patient receiving radiotherapy, it should have some special characteristics differentially from other four cell lines. Hence, a correlation analysis between the miR-139-5p expression and NR5A2 expression was performed in four ESCC cell lines except for KYSE-150. The result showed a moderate negative correlation between miR-139-5p and NR5A2 (R = −0.398 for NR5A2 mRNA, and R = −0.370 for NR5A2 protein, respectively).

The correlation of miR-139-5p and NR5A2 expression in esophageal tissues was analyzed using Pearson correlation. With the log-transformed relative expression data from tumor tissues and tissues adjacent to tumor, the Pearson correlation analysis showed a significantly negative correlation between miR-139-5p and NR5A2, as shown in Figure 6B (R = −0.284, P = 0.0002). However, no statistical differences were observed in the relative expression of NR5A2 between cancer tissues and adjacent controls.

## Discussion

The deregulation of miR-139-5p was observed to be a frequent event in ESCC and other types of cancer [13], [22], [23]. Kano et al. [13] identified the downregulation of miR-139-5p in ESCC tissues with a fold change of 0.175 by miRNA microarray. A recent study reported that the downregulation of miR-139-5p in hepatocelluar carcinoma (HCC) was significantly associated with poor prognosis of patients and identified features of metastatic tumors, including venous invasion, microsatellite formation, absence of tumor encapsulation, and reduced differentiation [23]. However, the functional role of miR-139-5p in ESCC has not been carefully investigated. The present study confirmed a 14.065-fold decrease expression of miR-139-5p in ESCC tissues compared with normal tissues, which was significantly associated with an increased risk for esophageal cancer. Meanwhile, the reduced expression of miR-139-5p in tumor patients was supposed to be linked with lymph node metastases. The results suggest that miR-139-5p functions as a tumor-suppressor gene involved in esophageal cancer development.

Analysis of the mRNA expression profiles revealed 354 differentially expressed genes, 89 upregulated genes, and 265 downregulated genes between miR-139-5p mimic-treated cells and NCs. The results imply that the alteration of miR-139-5p levels in ESCC cells may trigger major downstream events in carcinogenesis, which were classified by IPA. A number of processes or functions that were uniquely enriched were identified in the altered genes, including cellular movement, cellular growth and proliferation, cell death, and cancer. In this study, the cytobiology features regulated by miR-139-5p in the ESCC cell line were measured. The results show that miR-139-5p can suppress cell proliferation capability and participate in the retardation of G1/S phase transition during cell cycle progression. miR-139-5p can also induce the late apoptosis of ESCC cells in vitro and was further demonstrated to affect cell migratory activity and cell invasiveness. The experiments demonstrated the tumor suppressive features of miR-139-5p at the phenotype level, including its involvement in cell proliferation, cell cycle arrest, and cancer invasion, which were identified from the expression array at the gene level.

Thus, the upregulation of miR-139-5p may induce important cellular changes by targeting specific genes. According to the networks identified by IPA, NR5A2 was found to be an important factor potentially involved in the abovementioned processes. NR5A2 was identified as a potential miR-139-5p target owing to the conserved miR-139-5p binding site in the 3′UTR sequence of NR5A2 based on the prediction of TargetScan software. The NR5A2 protein was initially identified to be a critical cis-element of the enhancer II of hepatitis B virus gene expression and regulation and is responsible for the formation of protein complexes in combination with other hepatocyte transcription factors. NR5A2 was subsequently found to serve important functions in embryonic development. Recent studies suggested that NR5A2, also called LRH-1, can enhance proliferation and cell cycle progression in cancer cells [20], [24]. LRH-1 is known to induce the expression of cyclins D1 and E1, which are believed to be critical in cell cycle progression through the G1 phase and cell proliferation [17], [25]. LRH-1 suppression resulted in cell cycle arrest, which is mediated by the downregulation of cyclin E1 [26]. In addition, LRH-1 expression was found to be associated with invasive breast cancer. LRH-1 influenced cell migratory activity through actin remodeling, and its overexpression resulted in the post-translational cleavage of E-cadherin in conjunction with increased matrix metallopeptidase-9 expression, which has been suggested to contribute to increased invasive properties [19]. Baquié demonstrated the crucial function of NR5A2 in protecting human islets against stress-induced apoptosis [27]. Wang further demonstrated the induction of apoptosis in LRH-1 knockdown hepatocellular carcinoma cells [26]. The present study demonstrated that miR-139-5p downregulates NR5A2 protein and mRNA levels and acts as a post-transcriptional repressor by binding to specific 3′UTR sequences in the NR5A2 transcript. Cyclin E1 and MMP9 expression levels were reduced, indicating that these two downstream genes are regulated by NR5A2 and are suggested to participate in neoplasm proliferation and invasive process induced by miR-139-5p, respectively.

Given that miR-139-5p has been established to regulate NR5A2 expression by binding with the conserved site of NR5A2 in the 3′UTR region, the changes in NR5A2 expression levels are related to the expression changes in miR-139-5p, which was confirmed with four esophageal cancer cell lines. Furthermore, the correlation analysis results from tumor tissues shows a relationship during the physiological lower expression of miR-139-5p, whereas the results from adjacent normal tissues show a relationship during the physiological higher expression of miR-139-5p. Considering that miR-139-5p regulation of NR5A2 both in tumor tissues and in adjacent normal tissues operate under the same mechanism, the correlation of miR-139-5p and NR5A2 expression in tumor tissues would be similar to that in adjacent normal tissues. Our analysis showed no significant difference in the correlation coefficient between tumor tissues and adjacent normal tissues. Thus, all samples from tumor tissues and adjacent normal tissues were mixed to calculate the correlation coefficient of miR-139-5p and NR5A2. In this study, a significant negative correlation was found between miR-139-5p and NR5A2 expression in tumor tissues and in tissues adjacent to tumors, which suggests that miR-139-5p could affect oncogenic NR5A2 expression and is involved in the development and lymph node metastasis of esophageal cancer. Further analysis using TargetScan Release 6.2 predicted multiple miRNA target binding sites in the 3′UTR sequence of NR5A2, which meets the R-squared value of Pearson correlation analysis that showed the contribution of miR-139-5p to NR5A2 expression only accounted for approximately 8% in esophageal cancer patients. Considering that NR5A2 is regulated by miR-139-5p and showed no significant association with the risk for esophageal cancer, the results indicate that miR-139-5p is a more reliable indicator than NR5A2 for the early diagnosis of esophageal cancer.

## Materials and Methods

### Study Subjects and Ethics Statement

This study recruited 106 of patients with newly diagnosed, untreated esophageal cancer from Huaian County of Jiangsu Province, China. Patients were newly diagnosed with histologically confirmed primary cancer and previously untreated (no radiotherapy or chemotherapy) ESCC from October 2008 to December 2010. After participants provided their written informed consent to participate in this study, each subject was scheduled for an interview, and a structured questionnaire was administered by the interviewer to collect information on demographic data, clinical characteristics, and risk factors. Esophageal cancer tissues and adjacent normal tissues were collected from each subject during operation and then stored in RNAlocker reagent. The ethics committee, called “IRB of Southeast University Affiliated Zhongda Hospital” in Nanjing, China, approved this consent procedure. The population study was approved by the IRB of Southeast University Affiliated Zhongda Hospital in Nanjing, China. Odds ratios (OR) with 95% confidence intervals (95% CI) were calculated by conditional logistic regression to estimate the relative risk associated with miR-139-5p expression levels. Student’s t test was performed to evaluate whether miR-139-5p expression was associated with demographic and clinical characteristics at *α* = 0.05. Pearson correlation was used to analyze the relationship between miR-139-5p and NR5A2 expression.

### Cell Line and Culture

Five human ESCC cell lines were used in this study. EC109 and EC9706 were purchased from Shanghai Tiancheng Technology Co., Ltd. CaEs-17 were purchased from Nanjing KeyGen Biotech Co., Ltd. KYSE150 and TE1 were purchased from the Cell Center of Shanghai Institute of Life Science, Chinese Academy of Science. Cells were cultured in RPMI1640 medium (Invitrogen) supplemented with 10% heat-inactivated fetal bovine serum (FBS; Invitrogen) and 1% penicillin/streptomycin (Sigma-Aldrich). Cells were incubated in a humidified incubator at 37°C and 5% CO_2_.

### Construction of miR-139-5p Overexpressed Cell Model

Cells were transfected with either a miR-139-5p mimic or a scrambled NC (Guangzhou RiboBio Co., Ltd.) using the Lipofectamine™ RNAiMAX transfection reagent (Invitrogen), according to the manufacturer’s protocol. Briefly, 12 pmol of miR-139-5p mimic or NC was mixed with 2 µL of RNAiMAX reagent in 0.2 mL of OPTI-MEM (Invitrogen). After being added into a 12-well plate, the complex was incubated for 20 min at room temperature. Approximately 100,000 cells were then seeded into each well to a total volume of 1.2 mL. Oligonucleotide transfection efficiencies were determined by fluorescence microscopy using siR-Ribo™ Transfection Control (Cy3) (Guangzhou RiboBio Co., Ltd.). After incubation at 37°C and 5% CO_2_ for 48 h, cells were trypsinized for total RNA isolation.

### Isolation of Total RNA

Esophageal tissues were homogenized in 1 mL of TRIZOL reagent for every 30 mg to 50 mg of tissue. Total RNA was isolated from the homogenate according to the manufacturer’s instructions and then dissolved in RNase free water. Cell pellets were homogenized in 0.35 mL of guanidine thiocyanate reagent per 10^6^ cells. Total RNA samples were isolated with the RNeasy Mini Kit (Qiagen). RNA concentrations were determined spectrophotometrically by monitoring UV absorbance at 260 nm. Purity was assessed based on the absorbance ratio 260 nm/280 nm.

### qRT-PCR

Quantitative reverse transcription PCR analysis of miRNA and mRNA expression was carried out using a 7300 Real-Time PCR System (Applied Biosystems) using SYBR Green I dye. U6 small nuclear RNA and β-actin were used as internal controls to normalize RNA input for miRNA and mRNA assay, respectively. Briefly, 10 µL of reverse transcription (RT) reaction mixture contained 0.5 µg of total RNA, 5 nM RT primer, 500 µM dNTP mixture, 2 µL of 5×RT buffer, 10 U of Ribonuclease Inhibitor (Sigma), 100 U of MMLV (Promega), and RNase-free water. The RT reaction was performed as follows: 16°C for 30 min, followed by 42°C for 60 min, heated to 85°C for 5 min and subsequently stored at −20°C. Specific RT primers for miR-139-5p and U6 cDNA synthesis were purchased from Guangzhou RiboBio Co., Ltd. using miRNA-specific stem-loop RT primer design. Oligo dT_18_ (Invitrogen) was used as RT primer for NR5A2, MMP-9, cyclin D1, cyclin E1, and β-actin cDNA synthesis.

The real-time PCR was conducted in 96-well plates (Axygen) with a final volume of 20 µL using SYBR Green I dye. The PCR reaction components included the following: 1 µL of cDNA synthesized as above, 10 µL of 2×SYBR Green PCR Master Mix, and 0.6 µM of each pair of oligonucleotide primers. The following thermal cycling conditions were followed: 95°C for 5 min, followed by 40 cycles at 95°C for 15 s, and 60°C for 60 s. A dissociation curve analysis was added after the final PCR cycle to evaluate the presence of nonspecific PCR products and primer dimers. Specific PCR primers for miR-139-5p and U6 cDNA amplification were purchased from Guangzhou RiboBio Co., Ltd. Specific PCR primers for NR5A2, MMP-9, cyclin D1, cyclin E1, and β-actin cDNA amplification were synthesized by Invitrogen and the sequences were as follows. NR5A2∶5′- AGT ATC TCC TAG TCC CTT AAT T -3′ (sense) and 5′- TCG GTT CTG ACT TCT TTG GT -3′ (antisense). MMP-9∶5′- AGT TCC CGG AGT GAG TTG AA -3′ (sense) and 5′- CTC CAC TCC TCC CTT TCC TC -3′ (antisense). Cyclin D1∶5′- ACC AGC TCC TGT GCT GCG AAG TG -3′ (sense) and 5′- GAC GGC AGG ACC TCC TTC TGC ACA -3′ (antisense). Cyclin E1∶5′- AGC GGT AAG AAG CAG AGC AG -3′ (sense) and 5′- CGC TGC AAC AGA CAG AAG AG -3′ (antisense). Β-actin: 5′- ATC CGC AAA GAC CTG T -3′ (sense) and 5′- GGG TGT AAC GCA ACT AAG -3′ (antisense).

The template levels were compared between subjects using a comparative Ct method with separate tubes, as described elsewhere [28]. Briefly, the individual level of initial target cDNA was expressed as the difference in Ct between the target and internal control (ΔCt). All experiments were performed in triplicate. Paired Student’s t-test was performed to evaluate the differences in miR-139-5p expression between tumor tissue group and adjacent normal tissue group.

### Genome-wide Expression Microarray Analysis

The genomic expression level of total RNA samples from cells transfected with miR-139-5p mimic or NC was detected using Roche NimbleGen expression microarray. Signal hybridization and scan was performed by CapitalBio Corporation (Beijing, China). Fold changes in gene expression were determined with the normalized signal intensities of probes from miR-139-5p mimic treatment divided by the signal intensities from NC treatment. False discovery rate (FDR)-adjusted P values were determined for each gene. Transcripts were identified to be significantly differentially expressed if they fit the criteria of FDR <0.05 and fold change >|2.0|.

### Network Analysis of Identified Differential Transcripts

IPA software (Ingenuity Systems), which contained functional data based on peer reviewed publications [29], was used to analyze network and functional correlation with the above identified differentially expressed transcripts. The set of input genes matched with the Ingenuity Pathways Knowledge Base and their fold change values were used to identify hypothetical interaction models of genes. A Fisher’s exact test was performed to determine the likelihood that the genes are present in each identified network.

### Cytobiology Experiments

In this study, the tumor suppressive features of miR-139-5p were measured based on cell proliferation and cell cycle, migratory activity and invasion, and apoptosis. Analysis of variance (ANOVA) and Chi-square test were used to determine the statistical differences between different treatments in the following experiments at the 5% significance level.

#### Cell proliferation assay

The proliferation of EC109 was tested by EdU (5-ethynyl-2′-deoxyuridine) cell proliferation assay using Cell-Light™ EdU DNA Cell Proliferation Kit (Guangzhou RiboBio Co., Ltd.). Briefly, EC109 cells (1×10^4^) were seeded in each well of 96-well plates for transfection with miR-139-5p mimic or NC oligonucleotide. After incubation at 37°C and 5% CO_2_ for 48 h, cells were added with 50 µM EdU and incubated for another 2 h. Cells were then fixed with 4% paraformaldehyde and stained with Apollo® Dye Solution for proliferating cells. Nucleic acids in all cells were stained with Hoechst 33342. The cell proliferation rate was calculated according to the manufacturer’s instructions. Images were taken using a fluorescence microscope (Olympus FSX100). All experiments were performed in triplicate.

#### Cell cycle assay

Cell cycle was measured by flow cytometry. Briefly, EC109 cells (1×10^6^) treated with miR-139-5p mimic or NC oligonucleotide were trypsinized and fixed in 70% ethanol at 4°C overnight. The cells were then incubated in 200 µL of 1 g/L RNase A. After incubation for 30 min at 37°C, 1 mg/mL of propidium iodide (PI) solution was added to the cells, followed by 30 min of lightproof incubation at 4°C. Signals from each cell were captured with a flow cytometer (FACS Calibur, BD, USA). All experiments were performed in triplicate.

#### Apoptotic rate assay

EC109 cells (1×10^6^) with miR-139-5p mimic or NC oligonucleotide were trypsinized and resuspended in 500 µL of binding buffer with 5 µL of Annexin V-FITC and 5 µL of propidium iodide. After briefly incubating under light for 15 min at room temperature, the apoptotic rates of cells were detected using a flow cytometer (FACS Calibur, BD, USA). All experiments were performed in triplicate.

#### Cell migratory activity assay

The effect of miR-139-5p on cell migratory activity was detected by cell scratch experiment. EC109 cells (5×10^5^) were seeded in 6-well plates for transfection with miR-139-5p mimic or NC oligonucleotide. After incubation for 24 h, a linear wound was applied to the cells using a pipette tip. Cells were then rinsed with PBS and grown in RPMI1640 medium supplemented with 10% FBS for an additional 48 h. Wound healing was measured at five random fields at the time of wounding (time 0) and at 24 and 48 h after wounding with Image J software. Migration was evaluated with wound healing index. The wound healing index was defined as the ratio of (initial wound width-wound width after healing at specific time) divided by initial wound width. All experiments were performed in triplicate.

#### Cell invasive capability assays

The effect of miR-139-5p on cell invasive capability was detected using a 24-well transwell plate (8 µm pore size, Corning, New York, USA). Briefly, chamber inserts were coated with 200 mg/mL of matrigel and dried overnight under sterile conditions. EC109 cells (1×10^4^) treated with miR-139-5p mimic or NC were trypsinized and suspended in RPMI1640 medium supplemented with 10% FBS. The cell suspension was seeded into the top chamber, whereas RPMI1640 medium supplemented with 50% FBS was added to the lower chamber. After incubation for 24 h at 37°C, the top chambers were wiped with cotton wool to remove the noninvasive cells and subsequently fixed in 100% methanol for 10 min. After staining in hematoxylin-eosin solution, the invading cells on the underside of the membrane were counted at five random fields under a microscope (Olympus BX41). All experiments were performed in triplicate.

### Western Blot Analysis of Predicted Target NR5A2

Nuclear receptor subfamily 5, group A, member 2 (NR5A2) was predicted as a candidate target of miR-139-5p by the online TargetScan release 6.0 software (http://www.targetscan.org/). The protein levels of NR5A2 were determined in the EC109 cells transfected with miR-139-5p mimic or NC by Western blot analysis. Cellular proteins were extracted in RIPA buffer [50 mmol/L Tris-HCl, pH 8.0, 150 mmol/L NaCl, 1% (vol/vol) Nonidet P-40, 0.5% (wt/vol) sodium desoxycholate, and 0.1% (wt/vol) SDS] containing the complete protease inhibitor cocktail. Protein concentration was determined using the Bio-Rad protein assay (Bio-Rad). The isolated total proteins (40 µg) were used for SDS–PAGE separation. Western blot analysis was performed according to standard procedures as previously described [30]. Briefly, β-actin (45 KD) and NR5A2 (61 KD) were transferred to PVDF membrane at 250 mA for 1.5 h. After blocking overnight with 5% nonfat milk, primary mouse anti-human β-actin IgG (Sigma) and mouse anti-human NR5A2 IgG (Abcam) titers were optimized at 1∶5000 and 1∶1000, respectively, and then incubated with the membrane for 1.5 h at room temperature. HRP-goat anti-mouse IgG (Abcam) (dilution 1∶5000) was incubated for 1.5 h, followed by enhanced chemiluminescence detection (Pierce) according to the manufacturer’s protocol. The gray values of the stripes were captured using Chemilmager 5500 software, and the protein levels of the stripes were normalized based on the gray value of β-actin from NCs. Student’s t test was performed to evaluate the difference in NR5A2 protein between miR-139-5p-treated cells and control.

### Luciferase Reporter Assay

For confirmation of direct target binding, the wild type and mutant 3′UTR of NR5A2 identified by TargetScan was cloned into a pmiR-RB-Report™ dual luciferase Reporter gene plasmid vector (Guangzhou RiboBio Co., Ltd.). The 3′UTR region of candidate target gene was inserted downstream of the sequence of Renilla luciferase, which is designed for reporter fluorescence (Rluc). The sequence of firefly luciferase is constructed for calibration fluorescence (Luc) in the same vector as a stable internal reference to monitor the efficiency of plasmid expression. Dual luciferase reporter assay was performed according to standard procedures as previously described [30]. Briefly, EC109 cells were seeded into a 96-well plate with a concentration of 10 thousand cells per well in a total volume of 100 µL and incubated for 24 h. Cells were then co-transfected with miR-139-5p mimic or controls and 3′UTR vector. NC and miR-21* are the negative and non-specific controls of miR-139-5p, respectively. Vector control and mutant NR5A2 are the negative and non-target binding site controls of 3′UTR construct of NR5A2, respectively. After incubation for 24 h, the cells were used for luciferase assay. The signal from each well was read with Mithras LB 940 (Berthold Technologies). Luciferase signal ratio for Renilla over Firefly was calculated for each co-transfection treatment. All experiments were performed in triplicate. ANOVA was used to determine the statistical differences among different treatment groups at the 5% significance level.

### Statistical Analysis

All results were presented as mean ± SD. ANOVA, Chi-square test, and logistic regression analysis were used to determine the statistical differences at the 5% significance level. The relationship between miR-139-5p and NR5A2 expression was analyzed using the Pearson correlation. All statistical analyses mentioned above were performed using SPSS software (Version 13.0).

## Conclusions

In summary, miR-139-5p was identified as a tumor suppressor gene associated with the risk for esophageal cancer, and a novel mechanism of regulation of NR5A2 protein by miR-139-5p was reported. miR-139-5p was found to suppress cell proliferation and invasive capability of esophageal carcinoma cells and induce cell cycle arrest in the G0/G1 phase and in the late apoptosis of cancer cells by targeting oncogenic NR5A2. This study suggests that miR-139-5p suppresses growth and invasiveness in human ESCC by acting as a post-transcriptional repressor. Therefore, miR-139-5p can act as a potential biomarker for early diagnosis and prognosis of ESCC and a therapeutic target for ESCC.

## Supporting Information

Table S1 The differentially expressed transcripts influenced by miR-139-5p mimic in EC109 cells.
(XLS)

Table S2 Top 10 networks and functions of the differentially expressed transcripts influenced by miR-139-5p mimic in EC109 cells.
(XLS)
